# Association of maternal psychological distress with children with overweight/obesity in Ethiopia

**DOI:** 10.1111/cch.13057

**Published:** 2022-09-12

**Authors:** Sibhatu Biadgilign, Tennyson Mgutshini, Amare Deribew, Bizu Gelaye, Peter Memiah

**Affiliations:** ^1^ Department of Health Studies College of Human Science, University of South Africa Pretoria South Africa; ^2^ School of Public Health Saint Paul's Hospital Millennium Medical College Addis Ababa Ethiopia; ^3^ Nutrition International Addis Ababa Ethiopia; ^4^ Department of Epidemiology Harvard T.H. Chan School of Public Health Boston MA USA; ^5^ The Chester M. Pierce, MD Division of Global Psychiatry Massachusetts General Hospital Boston MA USA; ^6^ Division of Epidemiology and Prevention: Institute of Human Virology University of Maryland School of Medicine Baltimore MD USA

**Keywords:** common mental disorders, Ethiopia, obesity, overweight, psychological distress, SRQ‐20

## Abstract

**Background:**

Poor maternal mental health is a major risk factor for adverse offspring health outcomes, including overweight/obesity status. Maternal mental distress is highly prevalent and associated with parenting practices influencing child weight. To date, there is little information documented in Ethiopia on maternal mental distress and children with overweight/obesity status. This study examined the association between maternal mental distress and children with overweight/obesity among mother–child dyads in Addis Ababa, Ethiopia.

**Methods:**

An observational population‐based cross‐sectional study was conducted among mother–child dyads in representative samples in Addis Ababa, Ethiopia. Maternal mental distress was measured using the Self‐Reporting Questionnaire (SRQ)‐20. Child/adolescent overweight/obesity was defined as more than 1 SD above the median World Health Organization (WHO) growth reference. Multivariate logistic regression was used to estimate odds ratios (ORs) and 95% confidence intervals (CIs).

**Results:**

The prevalence of maternal mental distress and children with overweight/obesity was estimated to be 10.1% and 28.8%, respectively. After adjusting for confounders, including maternal education, maternal occupation, average monthly household income, maternal body mass index (BMI) and the number of household members/family size, maternal psychological distress was not associated with offspring overweight/obesity status (adjusted OR [aOR] = 0.54; 95% CI: 0.25, 1.14).

**Conclusions:**

There is no evidence of an association between maternal psychological distress and children with overweight/obesity. This lack of association might be attributable to our cross‐sectional study design. Future epidemiologic studies, particularly those using prospectively collected data, are warranted to examine better the effects of maternal psychological distress on offspring body weight.

**Key messages**

- Poor maternal mental health is a major risk factor for adverse offspring health outcomes including childhood obesity.
- As well, mental distress is prevalent and linked with parenting practices that influence child overweight and obesity.
- There is a low prevalence of maternal mental distress in our study.
- Presence of maternal psychological distress was not associated with children and adolescent overweight/obesity status.
- Other socio‐demographic variables might play a role for the development of children and adolescent overweight/obesity.

## 1 INTRODUCTION

Rising trends in children's and adolescent's obesity rates are major public health problems globally ((NCD‐RisC), 2017; Y. Wang & Beydoun, 2007; Y. Wang & Lim, 2012). The global prevalence of childhood overweight and obesity increased from 4.2% in 1990 to 6.7% in 2010, and the prevalence expected to increase with 9.1% or approximately 60 million children in 2020 with a relative increase of 36% from 2010 (Y. Wang & Lim, 2012). In 2010, 35 million children in low‐ and middle‐income countries (LMICs) were overweight and obese; 92 million were at risk of being overweight. Maternal mental health and early life environment are important factors linked to adverse health outcomes in the lifespan (Ramasubramanian et al., 2013), including the risk of childhood obesity (Hope et al., 2019). There is a well‐established body of literature documenting associations between maternal obesity with offspring overweight or obesity status (Eriksson et al., 2015; Gibson et al., 2007; Godfrey et al., 2017). In addition, mothers who experience higher perceived stress or are exposed to more stressors may have children at greater risk for obesity (Tate et al., 2015).

In the past 20 years, a great deal of attention has been given to parental influences on children with obesity. However, the parental factors in the obesogenic environment are still underdeveloped in the literature (Skouteris et al., 2011). Poor maternal mental health is one of the most common risk factors for adverse childhood health outcomes, including obesity (Goodman, 2007; Lampard et al., 2014). Maternal depression has been associated with parenting practices influencing child weight (Lampard et al., 2014). Other maternal risk factors associated with offspring overweight and obesity status include maternal overweight/obesity (Gibson et al., 2007; K. L. Whitaker et al., 2010; Yakovenko et al., 2019), being a single‐parent (single‐mother) family (Gibson et al., 2007), maternal pre‐pregnancy body mass index (BMI), family structure, and parenting quality (McConley et al., 2011), self‐esteem and anxiety (Benton et al., 2015; Lohman et al., 2009; McConley et al., 2011).

Maternal psychopathology (depression, anxiety, self‐esteem or body dissatisfaction) has also been linked to lifestyle risk factors contributing to childhood overweight and obesity. These include low physical activity levels, poor nutrition and increased sedentary behaviour (McConley et al., 2011; Taveras et al., 2010; Trost et al., 2003). Other suggests considering the various ways maternal psychosocial health may impact children's risk for overweight and obesity (Benton et al., 2015).

There is accumulating evidence that indicates high levels of parental depression are associated with high levels of BMI in children, and parental mental health may contribute to the children with obesity epidemic (Zarychta et al., 2020). To date, there is little information documented in developing countries, particularly in Ethiopia, on maternal mental distress and children with obesity status in the context of urbanization. This study aimed to examine the association between maternal mental distress and children with overweight/obesity in Ethiopia.

## 2 METHODS

### 2.1 Sample selection

The study design was an observational population‐based cross‐sectional study conducted in representative samples of mother and child dyads in the Addis Ababa city administration. The study data collection period was from May 2017 to July 2017. This study used data from 632 mother–child dyads enrolled in a larger study designed to assess the association of household food insecurity with overweight and obesity in children and adolescents. The details of the main study, including the study procedures, approach, sample size determination and sampling methods, are described elsewhere (Biadgilign et al., 2021).

### 2.2 Sampling procedure

Briefly, a multi‐stage sampling technique was carried out to identify the study population from selected sub‐cites. From each sub‐city, proportion to population sampling was applied to obtain the sample size. A simple random sampling method was used to choose districts in each sub‐city. One child was selected from single‐child households. For a household with multiple children, a random selection of one child was made using a lottery method.

### 2.3 Sample size calculation

The sample size was calculated using single‐proportion sample size formula. The parameters were as follows: 9.5% proportion of children who were overweight in the population (P) (Gebremichael & Chere, 2015), 95% confidence level, 3% margin of error for sampling and 80% power. This gave a sample size of 367. Then, by including 15% for the non‐response rate and design effect of 1.5, the total sample size was 634. Epi Info (Centers for Disease Control and Prevention, Atlanta, USA, 2010) statistical package was used to compute the sample size.

### 2.4 Study participants

The source population was mother–child pairs at the household level living in each sub‐city. In contrast, the study population was paired with sampled school‐aged children with their mothers present during the data collection period in selected sub‐cities and who fulfilled the inclusion criteria (i.e. those children who are living with their mothers, school‐aged children who are 5 to 18 years old, mothers who can respond to the interviewer and school‐aged children who lived in each of the sub‐cities for at least 5 years) to be included in the study.

### 2.5 Study setting

The study was conducted in Addis Ababa, considered as Africa's diplomatic venue. About 92 embassies and consular representatives are gathered in the city. Organizations like Africa Union (AU) and the UN Economic Commission for Africa (UNECA) have based their headquarters in Addis Ababa. The study area is divided into 10 sub‐cities, and each sub‐city is further divided into several small administrative units called Districts/Woreda. In Addis Ababa, which is the capital and largest city of Ethiopia, Yibeltal et al. (2013) indicate that overweight and obesity prevalence has been on the rise between 2000 and 2011, and among working adults in the city, a quarter of men (24.7%) and women (25.7%) are reported to be overweight and women were more likely to be obese (10.2%) compared with men (2.1%) (Tran et al., 2011).

### 2.6 Measurement

Common mental disorders (CMDs) include depression and anxiety such as generalized anxiety, panic, obsessive–compulsive and post‐traumatic stress, phobias or situations that make an individual vulnerable. Maternal mental distress was measured using the World Health Organization (WHO) recommended Self‐Reporting Questionnaire‐20 (SRQ‐20) and has been validated in Ethiopia (Alem et al., 1999; Hanlon et al., 2008, 2015; Kortmann & ten Horn, 1988). The SRQ‐20 measures several symptoms of depression, anxiety, panic and/or somatic symptoms experienced by the person. The tool has 20 questions. Each question was given a score of 0 or 1 depending on the binary ‘no’ or ‘yes’ responses. The scores were added to generate an overall SRQ‐20 scale, where higher scores indicate higher levels of maternal CMD and otherwise. In this study, a cut‐off of 6 was used to classify women with a low or high level of mental distress (Hanlon et al., 2008).

### 2.7 Questionnaire development

The questionnaires were developed after an in‐depth literature review and using standard scales for structure. The data were collected using a structured questionnaire originally developed in English. The content of the questionnaire included socio‐demographic characteristics (age and gender of the child, age of mother and educational and occupational status of mother), socio‐economic status, maternal CMD status (anxiety and depression) and nutritional status indicators based on anthropometric measurements. The questionnaire was translated to local languages and back‐translated to English by a translator who was blind to the original questionnaire.

### 2.8 Data collection procedure

Data were collected by trained data collectors using standardized, structured and pre‐tested questionnaires. This quantitative study was conducted by interviewing teams conducting the data collection process. Each team consisted of one supervisor, four female interviewers and two male interviewers. Height and weight measurements were conducted on school‐aged children in all selected households. Weight measurements were obtained using lightweight SECA mother–infant scales with a digital screen designed and manufactured under the guidance of the United Nations International Children's Emergency Fund (UNICEF). Height measurement was conducted using a measuring board while standing. The weight and height of each child were measured after calibrating to the nearest 0.1 kg and 0.1 cm. Height and weight measurements of children were converted into Z‐scores based on the WHO reference population considering their age and sex.

### 2.9 Maintaining and ensuring data quality

The data were well monitored at the time of data collection and entry level. Pre‐testing of the questionnaire on 10% of the final sample size was undertaken in a similar location before the actual data collection occurred. The study participants for the pre‐test were not included in the main study as they were not selected randomly. Iterations after the pilot informed the final version of the questionnaire used for the data collection process. The supervisors were trained in data quality control procedures and fieldwork co‐ordination. Several rounds of supervision were carried out by principal investigators to assess the quality of field operations and to ensure the proper listing of enumeration areas/households in each sub‐city. Adequate training was given to all supervisors and data collectors during data collection. Moreover, all activity was checked by the study's researchers with structured supervisory visits throughout the data collection period. All completed questionnaires were inspected for completeness and consistency during data management and analysis.

### 2.10 Covariates

We used conventional cut‐offs or classifications based on response distributions to code demographic variables. Other variables were categorized as age of children (5–9, 10–14 and 15–18 years), gender of children and adolescents (male and female), household head's gender (male and female), age group of mothers (<35, 35–49 and >49 years), mothers' education status (illiterate and literate), mothers' occupation (unemployed, private business and employed), marital status of the respondents (married and currently unmarried), maternal BMI (obesity and non‐obesity) and the number of people in the household/family size. Household monthly income was divided into tertiles and labelled as ‘high’, ‘medium’ and ‘low’ income categories.

### 2.11 Statistical analyses

Data were entered into Statistical Package for Social Science (SPSS) Version 21 and exported for analysis using STATA 15.0 (Stata Corporation, College Station, TX, USA) and WHO AnthroPlus software v1.02 (WHO, Geneva, Switzerland). The z‐score values for BMI‐for‐age (BAZ) of children and adolescents were generated relative to the WHO 2007 reference using the WHO AnthroPlus software (WHO, 2009). The analysis included descriptive analysis to characterize the study population and socio‐demographic characteristics. Means and standard deviations (SDs) were computed for continuous variables, and frequencies and percentages for categorical variables. The dependent variable used for the analysis was childhood overweight and/or obesity as a binary variable based on WHO growth reference (i.e. child/adolescent overweight/obesity was defined as more than 1 SD above the median WHO growth reference), and the exposure variables were socio‐economic status and socio‐demographic factors.

In this study, we examined the exposure of maternal CMD measured in relation to measuring maternal anxiety and depressive symptoms. Unadjusted, age‐adjusted and multivariable‐adjusted models were fit to assess the association between maternal CMD and childhood overweight/obesity. Adjustment was made for putative confounders identified a priori based on their relationship between maternal CMD and childhood overweight/obesity status. The confounders include age of the children and adolescents, maternal education, maternal occupation, average monthly household income, maternal BMI and number of household members/family size. We also assessed the odds of children and adolescent overweight/obesity across tertiles of CMD scores where tertiles were defined because of the distribution of the scores from the datasets. Odds ratio (OR) and its 95% confidence intervals (95% CIs) were used to interpret the study's findings. A value of *P* < 0.05 was considered statistically significant. This study followed the reporting requirements of the Strengthening the Reporting of Observational Studies in Epidemiology (STROBE) statement for reports of observational studies (von Elm et al., 2007) (Data S1).

## 3 RESULTS

A total of 632 children and adolescents–parent dyads were included in the study. The mean age of the household head was 45.0 (SD ± 8.81) years, and 63% of household heads were between the ages of 35 and 49. About 61.6% of mothers had formal education, whereas 33.4% of the mothers were unemployed. The mean (SD) household monthly income of participants (*n* = 525) was 7193.2 (±5324.6) Ethiopian Birr (1 USD ≈ 22.89 Birr [as of 10 May 2017]). About 48.4% of children were male and 51.1% were within the age group of 10–14 years. Children's mean (SD) age was 12.5 ± 2.96 years (Table 1). The prevalence of maternal mental distress and children with overweight/obesity was estimated to be 10.1% and 28.8%, respectively.

**TABLE 1:** Sample description: Parent and children and adolescents data in Addis Ababa, Ethiopia

Characteristics/variables	Categories	Total sample	Children with overweight/obesity		*P* value
		*N* (%)	Yes *n* (%)	No *n* (%)	
**Child characteristics**					
Children's age (years)^a^		12.51 ± 2.96	11.8 ± 2.79	12.8 ± 2.98	0.001
Age group of children and adolescents	5–9 years	125 (19.8)	46 (25.2)	79 (17.6)	0.006
	10–14 years	323 (51.1)	98 (53.9)	225 (50.0)	
	15–18 years	184 (29.1)	38 (20.9)	146 (32.4)	
Gender of children and adolescents	Male	306 (48.4)	85 (46.7)	221 (49.1)	0.583
	Female	326 (51.6)	97 (53.3)	229 (50.9)	
**Maternal characteristics**					
Household head's gender	Male	481 (76.1)	140 (76.9)	341 (75.8)	0.760
	Female	151 (23.9)	42 (23.1)	109 (24.2)	
Household head age (years)^a^		45.04 ± 8.81	45.8 ± 9.18	44.74 ± 8.65	0.082
Age group of mother	<35 years	50 (7.90)	16 (8.80)	34 (7.56)	0.713
	35–49 years	397 (62.8)	110 (60.4)	287 (63.8)	
	>49 years	185 (29.3)	56 (30.8)	129 (28.7)	
Maternal education	Illiterate	243 (38.4)	63 (34.6)	180 (40.0)	0.208
	Literate	389 (61.6)	119 (65.4)	270 (60.0)	
Maternal occupation	Unemployed	211 (33.4)	50 (27.5)	161 (35.8)	0.011
	Private business	126 (19.9)	30 (16.5)	96 (21.3)	
	Employed^b^	295 (46.7)	102 (56.0)	193 (42.9)	
Marital status	Married	532 (84.2)	153 (84.1)	379 (84.2)	0.961
	Currently unmarried	100 (15.8)	29 (15.9)	71 (15.8)	
Number of household members^a^		4.63 ± 1.41	4.75 ± 1.35	4.58 ± 1.43	0.115
Average monthly household income^c^	High	176 (33.5)	41 (28.7)	135 (35.4)	0.039
	Medium	194 (37.0)	48 (33.5)	146 (38.2)	
	Low	155 (29.5)	54 (37.8)	101 (26.4)	
Maternal BMI	Obesity	242 (38.3)	81 (44.8)	161 (35.8)	0.036
	Non‐obesity	389 (61.7)	100 (55.2)	289 (64.2)	
Maternal common mental disorders (CMDs)	No mental distress	567 (89.9)	170 (93.4)	397 (88.2)	0.032
	Presence of mental distress	65 (10.1)	12 (6.6)	53 (11.8)	
Total		632	182 (28.8%)	450 (71.2%)	

Abbreviation: BMI, body mass index.

^a^ Mean ± SD (standard deviation).

^b^ Employed includes government and NGO employee.

^c^ Income was categorized into tertiles as low, medium and high.

In the unadjusted model, maternal psychological distress was associated with children and adolescent overweight/obesity status (crude OR [cOR] = 0.48; 95% CI: 0.25, 0.95) (Model 1). Furthermore, maternal psychological distress was associated with children and adolescent overweight/obesity status (cOR = 0.45; 95% CI: 0.23, 0.89) even after adjusting for the age of the children and adolescents (Model 2). After further adjustment for confounders, including maternal education, maternal occupation, average monthly household income, maternal BMI and the number of household members/family size, maternal psychological distress was not associated with children and adolescent overweight/obesity status (adjusted OR [aOR] = 0.54; 95% CI: 0.25, 1.14) (Model 3) (see Table 2).

**TABLE 2:** Final odds ratios for overweight/obesity in children and adolescents with maternal common mental disorders

Characteristics/variables	Categories	Model 1 Unadjusted/crude OR (95% CI)^a^	Model 2 Adjusted OR (95% CI)^b^	Model 3 Adjusted OR (95% CI)^c^	*P* value
Maternal common mental disorders (CMDs)	No mental distress	Reference	Reference	Reference	0.096
	Presence of mental distress	0.48 (0.25, 0.95)	0.45 (0.23, 0.89)	0.54 (0.25, 1.14)	
CMD score	Lowest tertile	Reference	Reference	Reference	
	Middle tertile	1.40 (0.92, 2.15)	1.36 (0.89, 2.10)	1.42 (0.86, 2.35)	0.173
	Highest tertile	0.74 (0.48, 1.14)	0.71 (0.46, 1.10)	0.78 (0.47, 1.29)	0.329
CMD score (continuous)		0.93 (0.88, 0.99)	0.93 (0.87, 0.99)	0.94 (0.88, 1.00)	0.076

Abbreviations: CI, confidence interval; OR, odds ratio.

^a^ Unadjusted model.

^b^ Adjusted for age of the children and adolescents.

^c^ Further adjusted for maternal education, maternal occupation, average monthly household income, maternal body mass index and number of household members/family size.

## 4 DISCUSSION

This cross‐sectional study investigated associations between maternal psychological distress and childhood overweight. Our study did not find a significant relationship between maternal mental disorder and overweight/obesity among children and adolescents. Similar findings were observed from other studies that found no indication of an aetiological relationship between maternal distress and the development of childhood (Ajslev et al., 2010; Grote et al., 2010; Guxens et al., 2013; L. Wang et al., 2013).

A recent systematic review by Lampard et al. (2014) found mixed results from studies investigating the relationship between maternal depression and children with overweight/obesity, particularly for episodic depression. However, chronic depression was associated with a greater risk of child overweight status (Lampard et al., 2014). In a study from low‐income urban families in the United States with 401 mother–child dyads, others with moderate to severe depressive symptoms (aOR: 2.62; 95% CI: 1.02, 6.70) were more likely to have children with overweight and obesity than mothers without depressive symptoms (Gross et al., 2013). Other evidence in the same study revealed that children of mildly depressed mothers were more likely to consume sweetened drinks and to eat out at restaurants and were less likely to eat breakfast than children of non‐depressed mothers. Mothers with depressive symptoms were less likely to set limits, use food as a reward, restrict their child's intake and model healthy eating than non‐depressed mothers (Gross et al., 2013). Childhood overweight and maternal distress were coexisting, and mothers of obese youth reported significantly greater psychological distress, higher family conflict and more mealtime challenges (Zeller et al., 2007), and maternal depression as parenting behaviours is a single most important component of family ecology to consider for healthy child weight field (Lovejoy et al., 2000).

L. Wang et al. in their study (2013) found that as compared with children of mothers without depression at any of the three‐time points (1, 24 and 36 months of age after birth), children of mothers with depression at all three‐time points were more likely (aOR: 1.69) to be overweight after adjusting for other child characteristics and children of mothers with depression at all three‐time points were two times (aOR: 2.13) more likely to be overweight after controlling for maternal characteristics.

The basic concept for increasing tendency children with overweight/obesity mainly relies on programmes that have targeted parenting practices, such as child‐feeding strategies, and missed the maternal psychological profile like parents' anxiety, depression and emotional distress (Mitchell et al., 2013). Apparently, the existing literature documented that distressed mothers are at increased risk of neglecting their children, that attempted maternal psychological well‐being after delivery may also be a possible determinant of childhood overweight (Mitchell et al., 2013) and also that parental neglect greatly increased the risk in comparison with harmonious support and dirty and neglected children had a much greater risk of adult obesity; maternal self‐report of neglectful behaviour and family structure (biological or other parents and number of siblings) did not significantly affect the risk of adult obesity (Knutson et al., 2010; Lissau & Sørensen, 1994; R. C. Whitaker et al., 2007). This is supported by the fact that neglect hypothesis found associations between neglect in school age and overweight in early adulthood (Lissau & Sørensen, 1994).

According to the theoretical developments, maternal psychological distress may influence the stress level of the infant through direct and indirect mechanisms of behavioural and hormonal origin. The hormonal influence may be due to changes in stress hormones, which could be mediated through infant breastfeeding. By this means, maternal distress can contribute to the metabolism and growth of the infant through changes in hormonal and endocrine responses, thus leading to overweight and obesity (Ajslev et al., 2010). Some investigators suggested that the complex regulation and/or dysregulation of the maternal hypothalamic–pituitary–adrenal (HPA) axis during maternal psychological distress in both child and adulthood with a corresponding increase of maternal cortisol levels where adaptational changes in behaviour and metabolism that distress changes in the early perinatal period to be of importance for later development of obesity (Charmandari et al., 2003, 2005; Diego et al., 2006) and infant feeding practice or the mothers handling of the child could affect the early programming of the HPA axis and thereby induce stress and appetite regulation (Oberlander et al., 2008). The study has some strengths and limitations to be mentioned. As it is a population‐based study with a large sample size, the study is valid to draw a solid conclusion.

Furthermore, the study considers some adjustments for children and maternal characteristics and other variables into consideration related to children with overweight/obesity status. Although our study has several strengths, we note some limitations. First, our outcome variable, maternal mental distress, relies on recall and, therefore, there could be a likelihood of recall bias. Whilst we mitigate this concern by using a composite score for different common mental disorders, recall bias cannot be ruled out. Second, we do not consider retrospective data of the children and their mothers, which is an important topic for future research. Finally, the major sources of limitation are temporary and biological plausibility. In this study, we explored how maternal mental distress is related to offspring obesity status. As such, the source of obesity for the offspring could be multifaced. Given this limitation, our study can be viewed as generating a hypothesis that needs to be fully explored in future longitudinally designed epidemiologic studies.

In conclusion, in our study, there was no association between maternal psychological distress and childhood overweight/obesity. There is a low prevalence of maternal mental distress in our study. Interventions for children with overweight/obesity shall focus on other factors. Future epidemiologic studies, particularly those using prospectively collected data, will be recommended to understand and examine the determinants and nexus of maternal psychological distress impact on children overweight/obesity. In addition, future studies need to assess the effect of other psychiatric disorders on children with overweight/obesity in the country and across other continents.

## CONFLICT OF INTEREST

The authors declare that they have no conflict of interest.

## ETHICAL CONSIDERATION

Ethical clearance was obtained from the Department of Health Studies, University of South Africa Ethical Clearance Committee for Research on Human Subjects (HSHDC/575/2016) and Addis Ababa City Administration Health Bureau (A/A/H/B/3542/227). Official letters of co‐operation from the above organizations were given to respective sub‐cities and district administrators. Verbal informed consent was obtained from study participants. Here, for those children under the age of 18 years, verbal informed consent was obtained from their parents or caregivers. Assent was obtained from each children and adolescent.

## AUTHOR CONTRIBUTIONS

SB and TM conceived and designed the study, analysed the data, interpreted the data and wrote, reviewed and edited the manuscript. AD, BG and PM provided inputs for the analytical plans, critically reviewed the study and participated in data interpretation and write‐up of the manuscript. All authors critically reviewed and approved the manuscript and meet ICMJE criteria for authorship.

## Supporting information


**Data S1.** Supporting Information

## Data Availability

The data that support the findings of this study are available from the corresponding author upon reasonable request.
